# Integrating In Vitro Dissolution and Physiologically Based Pharmacokinetic Modeling for Generic Drug Development: Evaluation of Amorphous Solid Dispersion Formulations for Tacrolimus

**DOI:** 10.3390/pharmaceutics17020227

**Published:** 2025-02-10

**Authors:** Evangelos Karakitsios, Maria-Faidra-Galini Angelerou, Iasonas Kapralos, Georgia Tsakiridou, Lida Kalantzi, Aristides Dokoumetzidis

**Affiliations:** 1 Department of Pharmacy, National and Kapodistrian University of Athens, 15771 Athens, Greece; evanskarak@pharm.uoa.gr (E.K.); ikapralo@pharm.uoa.gr (I.K.); 2 Pharmathen SA, 15125 Athens, Greece; maggelerou@pharmathen.com (M.-F.-G.A.); gtsakiridou@pharmathen.com (G.T.); lkalantzi@pharmathen.com (L.K.)

**Keywords:** physiologically based biopharmaceutics model, amorphous solid dispersion, biorelevant dissolution test

## Abstract

**Objectives**: Tacrolimus, a Biopharmaceutics Classification System (BCS) class II drug, is widely used for transplant patients to prevent graft rejection. To enhance its bioavailability, amorphous solid dispersion (ASD) formulations were developed and evaluated. The release properties of several ASD-based tacrolimus formulations were studied using an in-house USP IV dissolution method. **Methods**: The pharmacokinetics of a promising test product were compared with the commercially available Advagraf^®^ in a pilot clinical bioequivalence study with 12 healthy subjects. A previously published PBPK model for tacrolimus was validated using in vivo data and then applied to predict the human pharmacokinetics of several ASD-based tacrolimus formulations. **Results**: This study compares the pharmacokinetic (PK) parameters—AUC, Cmax, and Tmax—of Advagraf^®^ and a test formulation using two methodologies: one incorporating the dissolution profile directly into the PBPK model and the other utilizing the DLM approach. The results show that both methods provided accurate predictions for Cmax and Tmax, with the dissolution profile approach underestimating AUC slightly, while the DLM method predicted AUC adequately. Sensitivity analysis refining the DLM scalars in the Ileum and Colon led to optimized predictions of PK parameters. Furthermore, this study explores the use of PBPK modeling to predict in vivo behavior for additional tacrolimus formulations, highlighting the influence of formulation composition, such as the inclusion of Eudragit-S100, on dissolution profiles and bioavailability. **Conclusions**: This study evaluates formulations with different compositions and manufacturing characteristics; key factors that could influence their performance in the body were identified. These insights—spanning qualitative, quantitative, and manufacturing aspects—can greatly simplify the development of generic drugs, offering strong evidence of the critical role that physiologically based pharmacokinetic (PBPK) modeling can play in the early phases of generic drug development, especially in designing and assessing biopredictive dissolution methods.

## 1. Introduction

Formulation and process development in the generic pharmaceutical industry are challenging due to the need for rapid market entry, inherent variability in processes and materials, and high development costs. Given these challenges, achieving time and cost efficiency is crucial, along with finding strategies to expedite product market entry.

Generic development is particularly difficult for poorly soluble drugs formulated into complex matrices using intricate manufacturing processes. However, advancements in in vitro dissolution testing have provided valuable insights into gastrointestinal processes, enhancing the correlation between in vitro data and absorption behavior through sophisticated equipment and biorelevant media. Additionally, physiologically based pharmacokinetic (PBPK) modeling has become essential for understanding in vivo behavior, offering insights into the characteristics and performance of various formulations.

In recent years, the integration of in vitro dissolution methods with physiologically based pharmacokinetic (PBPK) modeling has become an increasingly powerful strategy in pharmaceutical development, offering deeper insights into drug absorption mechanisms and facilitating more accurate predictions of in vivo performance. This combined approach is particularly advantageous for addressing the challenges associated with poorly soluble weakly basic drugs, which often exhibit complex dissolution and absorption behaviors [1]. By using PBPK models in conjunction with biorelevant dissolution testing, researchers can simulate in vivo conditions, accounting for factors such as gastric emptying, food effects, disease states, population variations, and drug transporters. Several studies have demonstrated the efficacy of this integrated approach [2]. For example, the development of a clinically relevant specification (CRS) using PBPK modeling successfully differentiated bioequivalent and non-bioequivalent batches of drug products, optimizing formulation control and ensuring consistent pharmacokinetic outcomes (e.g., for poorly soluble drugs such as isosorbide mononitrate) [3,4]. In the same line, the use of PBPK models alongside biorelevant dissolution data for a poorly soluble drug candidate, T2CP, formulations enabled accurate predictions of oral bioavailability, closely matching the observed plasma profiles in animal studies, thereby highlighting the utility of these tools in forecasting the in vivo performance of enhanced formulations [5]. In a different study involving metronidazole, it was demonstrated that despite significant differences in in vitro dissolution profiles, PBPK models could accurately predict bioequivalence, reinforcing the potential for biowaivers in certain drug classes [6]. Moreover, this integrated strategy was crucial in understanding the behavior of drugs like Irbesartan, where multi-compartmental biorelevant dissolution models and PBPK simulations provided a more mechanistic understanding of supersaturation and precipitation in the intestine, offering insights into the drug’s absorption behavior and improving predictions of plasma exposure [7]. Lastly, the impact of drug–drug interactions, such as those involving acid-reducing agents (ARAs) and dipyridamole, was also effectively modeled using this approach, where biorelevant media in conjunction with PBPK modeling successfully simulated the effect of ARAs on drug pharmacokinetics, underlining the predictive power of this integrated methodology [8].

For tacrolimus, PBPK has been extensively employed to gain insights into different aspects of product development. For example, in the early stages of development, PBPK has been utilized to optimize the dissolution method and evaluate biorelevant dissolution media as well as in vivo drug performance [9]. In addition to this, PBPK combined with dissolution testing was used to investigate the impact of drug crystallinity in amorphous tacrolimus capsules [10]. In another study, Gao et al. employed PBPK for a subcutaneously administered (S.C.) tacrolimus injectable formulation to elucidate the contributions of in vitro drug release, drug degradation, diffusion rate, and lymphatic transport on the absorption process [11]. Moreover, PBPK for tacrolimus has been applied to study the intestinal first-pass metabolism of CYP3A substrates with high intestinal extraction [12]. Furthermore, tacrolimus PBPK has been used to provide valuable pharmacokinetic information in different populations such as renal and heart transplant patients and pediatric populations [13,14,15]. Finally, PBPK has been instrumental in investigating potential drug–drug interactions involving tacrolimus [16].

In previously published work by Tsakiridou et al., tacrolimus, a poorly soluble model drug, was formulated into amorphous solid dispersion formulations [17,18]. In that study, different dissolution methods were developed to generate in vitro profiles for the lead formulation alongside the reference product, Advagraf^®^. The lead formulation, along with Advagraf^®^, was evaluated in a comparative bioavailability study. Among the different dissolution methods employed, the authors concluded that the method using compendial apparatus IV (in open loop mode) with biorelevant media to simulate physiologically relevant residence time was successful. This method was effective in screening for product-related differences in tacrolimus’ early exposure after the administration of amorphous solid dispersions with modified release characteristics in the fasted state.

In the present work, a PBPK model was developed for tacrolimus to demonstrate how a biopredictive method can be established in the early stages of generic development. Herein, a PBPK model is developed and the previously biopredictive dissolution data for the test and reference formulations are introduced to provide predictions for in vivo profiles. Moreover, different formulation candidates are screened through PBPK modeling to provide significant insights into the identification of the most promising candidate for generic drug development. This work further demonstrates how PBPK modeling can effectively streamline the market entry of generic products in a time- and cost-efficient way.

## 2. Materials and Methods

### 2.1. Materials

Ethylcellulose (EC, Ethocel STD 10 Premium) was purchased from Colorcon Inc.^®^ (Harleysville, PA, USA). From this company, we also purchased the polyethylene glycol, polyvinyl acetate, the polyvinylcaprolactame-based graft copolymer (Soluplus^®^), and the poly(ethylene oxide)-poly(propylene oxide)-poly(ethyelene oxide) triblock copolymer (Poloxamer-1). The rest of the materials used were described in a previously published work [17].

### 2.2. Formulation Preparation

Eight formulations were prepared previously, and their compositions are presented in Table 1. The preparation method was extensively described in a previously published work [17]. Further to this preparation method, the mode of surfactant incorporation was differentiated between physical mixing (extra-granular) or incorporated during the solid dispersion process (intra-granular). Specifically, the surfactants were incorporated via two methods: (1) physical mixing with the already spray-dried tacrolimus–polymer ASDs and (2) during the spray-drying step, where the tacrolimus, polymer, and surfactant were dissolved in the same solvent and spray-dried together.

### 2.3. In Vitro Dissolution Testing

In vitro dissolution testing has been conducted for all 8 formulations of Table 1 and Advagraf^®^ using the compendial apparatus IV with biorelevant media. The procedure performed and the analytical method are described in detail in a previously published work [17]. Briefly, the apparatus was used in the open-loop mode, where new dissolution medium was continuously introduced into the system, and the temperature of the water bath was set to (37 ± 0.5) °C. The dissolution media were introduced into the cells in a gradient, with a flow rate as shown in Table 2. The dissolution media were prepared to mimic the in vivo gastrointestinal compartment fluid, considering the physiological pH, buffer capacity, bile components, dietary lipids, lipid digestion products, and osmolality. Level III Fasted State Simulated Gastric Fluid (FaSSGF), Level II Fasted State Simulated Intestinal Fluids (FaSSIF-V2, FaSSIFV2midgut, and SIFIleum-V2), and Level II Fasted State Simulated Colonic Fluid (FaSSCoF) [19] were prepared with FaSSIF/FeSSIF/FaSSGF, FaSSIF-V2, and FaSSCoF powders (Biorelevant, London, UK), according to the instructions on the biorelevant.com website. The dissolution media, duration of exposure, and flow rates during the experiment were taken as recently described by Reppas et al. (2020) [20] Each medium was run for physiologically relevant residence times, as summarized in Table 2.

### 2.4. Clinical Data

The pharmacokinetics of formulation TAC1I8 (denoted as the test formulation) were studied in comparison with two commercially available products, Advagraf^®^ and Envarsus^®^, in a phase 1, crossover, 3-way, relative bioavailability clinical study in 12 healthy volunteers. This study was an open-label, balanced, randomized, single-dose, three-treatment, three-sequence, three-period, three-way, crossover, exploratory bioequivalence study. Each subject received one 5 mg prolonged-release hard capsule of Advagraf^®^ and one 5 mg tablet of the test formulation, as per the randomized schedule. The dose units were administered orally to each subject in a sitting posture. Twelve healthy, male, adult (23–43 years of age), non-smoker Asian volunteers participated in this study. The detailed specifics of this study are extensively presented in a previously published work [17].

### 2.5. PBPK Model Building

#### 2.5.1. Software Tools and PBPK Model Development

The SimCyp Simulator (Version 23, Certara, Sheffield, UK) was utilized to develop a PBPK model for the in vitro–in vivo extrapolation of tacrolimus amorphοus solid dispersion formulations. The parameter values to setup the SimCyp compound file for tacrolimus were taken from the literature, namely from Van der Veken et al., 2023 [15], and are listed in the Supplementary Materials Table S1. Briefly, a concentration-dependent blood-to-plasma partitioning was included, considering the extensive distribution of the drug into red blood cells. It is also noted that in our current analysis, whole-blood concentrations are used as observed and data are simulated to avoid any confounding effects. Regarding distribution, a minimal PBPK model was utilized, which is available in SimCyp and implements 2-compartment disposition kinetics. Moreover, the advanced dissolution, absorption, and metabolism (ADAM) model in SimCyp was used to describe absorption, while two metabolic pathways with CYP3A4 and CYP3A5 were also utilized. It is noted that within ADAM, the gastrointestinal tract is composed of 9 compartments, namely Stomach, Duodenum, Jejunum I, Jejunum II, Ileum I, Ileum II, Ileum III, Ileum IV, and Colon.

#### 2.5.2. In Vitro–In Vivo Extrapolation Approach

Regarding the dissolution model used in SimCyp, two different approaches were tested within ADAM to explore their impact on the in vivo PK prediction of the Advagraf^®^ formulation. In the first one, the direct input of the measured, biorelevant, in vitro USP IV dissolution data was exploited to simulate the in vivo PK concentration–time profile. Dissolution data were obtained from the Ph.D. dissertation of Tsakiridou (2020), who used compendial apparatus IV for the investigation of the dissolution behavior of Advagraf^®^ (5 mg/capsule) and the test formulation (5 mg/tablet).

In the second approach, the dissolution profiles of both Advagraf^®^ and the test product were used to estimate parameter values for the mechanistic DLM implemented in SimCyp’s SIVA toolkit and, more specifically, the DLM scalar parameter for each region of the GI. Since SIVA accepts only 3 media per experiment in USP IV apparatus and the available profiles had 5 media, grouping was carried out. Grouping was different between the Advagraf^®^ and the test formulations since the profiles were quite different and, more specifically, the test formulation reached 100% at about 2.5 h and therefore there was no information for dissolution in the Ileum and Colon, while Advagraf^®^ only reached about 50% of release at the end of the experiment at 6 h. For this reason, the test product’s parameter values for Ileum and Colon were handled by a sensitivity analysis.

Regarding the composition and physicochemical characteristics of the media utilized in SIVA, FaSSGF and FaSSIF-V2 were included within the toolkit. For the remaining three media, Level II biorelevant media were employed to simulate the environments of the middle and lower small intestine, as well as the ascending Colon in the fasted state, in accordance with the methodology described by Markopoulos et al. (2015) [19]. Specifically, a maleate buffer, derived from diprotic maleic acid (pKa_1_ = 1.83, pKa_2_ = 5.99), was employed with total buffer concentrations of 19.3 mM, 52.8 mM, and 75.8 mM for FaSSIF-V2 midgut, SIF Ileum-V2, and FaSSCoF, respectively. The pH of each medium was adjusted as follows: 6.8 for FaSSIF-V2 midgut, 7.5 for SIF Ileum-V2, and 7.8 for FaSSCoF. Furthermore, the sodium taurocholate concentration was used as a surrogate for the surfactant concentration in these media. Accordingly, surfactant concentrations of 1.5 mM, 0.8 mM, and 0 mM were employed for the midgut, distal Ileum, and ascending Colon, respectively.

The cumulative percentage dissolved was used as the input of the mechanistic absorption model ADAM, implemented in the SimCyp Simulator.

#### 2.5.3. Model Performance

To evaluate the PBPK model, PK data from a comparative bioavailability study were employed to compare the tacrolimus formulation (test product) with the reference product Advagraf^®^. To assess model performance in simulating the clinical data, a visual check was supplemented by numerical PK metrics, namely mean area under the concentration–time curve (AUC), Cmax, which is the highest measured concentration in vivo, with tmax being the time at which this concentration Cmax is reached. These PK parameters were compared to the respective observed ones. The system parameters were adjusted in the SimCyp Simulator to match the demographics of the actual study participants. Throughout the modeling assessment, a 2-fold error, defined as the ratio of the mean upper to the mean lower value for each parameter, was used as threshold for acceptable model performance, while a 1.25-fold error indicated good model performance.

## 3. Results and Discussion

### 3.1. Dissolution Modeling

Regarding the dissolution model used in SimCyp, as mentioned in the Methods Section, two approaches were employed. In the first approach, the direct input of measured, cumulative, in vitro dissolution data was exploited as the input to simulate the in vivo PK concentration–time profile. For the second approach, the mechanistic Diffusion Layer Model (DLM), built within SimCyp, was utilized to simulate such profiles. The DLM is a mechanistic model, and its parameters can be set from the properties of the compound. However, some may need to be estimated from the in vitro dissolution data. For this purpose, the SIVA toolkit, provided in SimCyp, allows us to estimate any model parameter. In our case, the parameter of the model was the so-called DLM scalar, while other model parameters took values found in Table S1.

More specifically, in the case of the Advagraf^®^ formulation, a DLM scalar value, for each dissolution medium, was optimized in SIVA and, finally, these five scalars were used as the input within the SimCyp Simulator. It is noted that in SIVA, only three media are permitted to be used as the input each time and, consequently, only three DLM scalars can be optimized on every occasion, not allowing all five scalars to be optimized concurrently. To overcome this limitation, initially, the three first dissolution media were utilized, namely FaSSGF, FaSSIF-V2, and FaSSIF-V2 midgut, to obtain the three respective scalars. The first scalar was used as the input within SimCyp in the Stomach, the second in the Duodenum, and the third one in Jejunum I and II. In addition, the immediate release formulation was selected within SimCyp.

Afterwards, to obtain the DLM scalar related to SIF Ileum-V2, the first two media (FaSSGF and FaSSIF-V2) were grouped together, while the two remaining media that were used in SIVA were FaSSIF-V2 midgut and SIF Ileum-V2. In this way, the first two DLM scalars with regard to the first dissolution media (first medium: FaSSGF and FaSSIF-V2; second medium: FaSSIF-V2 midgut) were disregarded, while the third DLM scalar, concerning the third medium SIF Ileum-V2, was retained and used as the input in SimCyp in all the Ileum compartments (Ileum I to IV). This method provided a good approximation of this final DLM scalar to overcome the PBPK platform’s limitation of permitting only three dissolution media on every occasion. The same approach was also taken for approximating the DLM scalar regarding the final dissolution medium FaSSCoF and used as the input in the Colon compartment in the SimCyp Simulator (first medium: FaSSIF-V2; second medium: SIF Ileum-V2; third medium: FaSSCoF). Ultimately, the impact of different approaches on the in vivo PK prediction was assessed by comparing simulations using the DLM scalars to simulations using the direct input of measured, biorelevant, in vitro USP IV dissolution data for Advagraf^®^.

In Figure S1 of the Supplementary Materials Section, the DLM scalar values for Advagraf^®^ are shown together with the corresponding fitted profiles.

Moreover, concerning the application of the second method, the SIVA model, to the test formulation, the DLM scalar parameter values were estimated for the first three dissolution media and used as the input for the Stomach, Duodenum, and Jejunum compartments. However, since the cumulative percentage dissolved reached 100% before the introduction of SIF Ileum-V2 in the in vitro biorelevant USP IV dissolution experiment, DLM scalars could not be estimated for the Ileum and Colon compartments. Hence, sensitivity analysis was performed for these two parameters in the SimCyp Simulator, comparing the simulated PK profiles to the in vivo observed data in healthy volunteers after the administration of the test formulation.

In Figure S2 of the Supplementary Materials Section, the DLM scalar values for Stomach, Duodenum, and Jejunum are shown for the test product. The results of the sensitivity analysis for Ileum and Colon are shown in the next section.

### 3.2. Comparison of IVIVE Predictions to the Clinical Study

Table 3 presents a comparison of the observed pharmacokinetic (PK) parameters—AUC, Cmax, and Tmax—calculated from the clinical study for both Advagraf^®^ and the test formulation against their respective simulated values. The simulations were conducted using two previously described methodologies: one employing the dissolution profile as the input and the other utilizing DLM. For Advagraf^®^, the results obtained from both methodologies were comparable. In the first approach, where the dissolution profiles were directly incorporated into the PBPK model, the predicted Cmax and Tmax values exhibited good concordance with the observed data, underscoring the high accuracy of the absorption prediction. However, the predicted AUC value was underestimated, with a fold error of 1.54, indicating the satisfactory, although not optimal, prediction of drug exposure. In contrast, the second approach, utilizing the immediate-release profile, demonstrated good agreement for Cmax and Tmax predictions, with fold errors of 1.26 and 1.29, respectively. The AUC prediction was highly accurate, with a fold error of 1.06, signifying an excellent prediction of overall drug exposure. Concerning the test formulation, both approaches yielded highly accurate predictions, with all the predicted PK parameters deviating by less than 1.17-fold from their observed counterparts. It is also noteworthy that the variability of the predicted AUC and Cmax values was greater than that of the observed values for both formulations in all cases, as evidenced by higher standard deviations. In contrast, the variability in the Tmax predictions was smaller compared to the observed data.

In addition, as mentioned previously, a sensitivity analysis was conducted to refine the prediction of the in vivo PK profile for the test formulation, using the immediate-release approach by adjusting the DLM scalars due to the SIVA toolkit limitations. The results of the sensitivity analysis, summarized in Table 4, revealed that increasing the DLM scalar in the Ileum (from 0.005 to 0.5) results in a reduction in the AUC and Tmax values, while the Cmax values remained largely unaffected. On the other hand, increasing the DLM scalar in the Colon (from 0.05 to 5.0) leads to a modest increase in the Cmax values, with a negligible impact on AUC and Tmax. The parameters ultimately incorporated into the SimCyp model were DLM scalars of 0.05 for Ileum compartments (I–IV) and 0.5 for the Colon. It is noted that these values, based on our analysis, should also be obtained with different in vitro USP IV dissolution experiments, utilizing the incorporation of SIF Ileum-V2 and FaSSCoF media before 100% release of the drug. However, this was not experimentally performed and cannot be validated.

**Figure 1:**
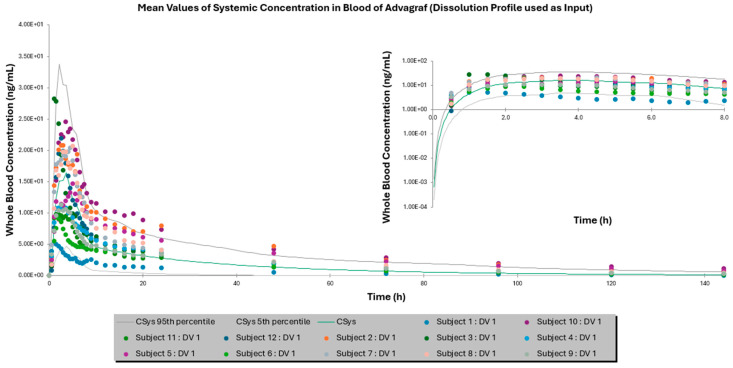
Mean whole-blood in vivo concentration versus time graph of Advagraf^®^ after the administration of a single dose for 144 h in the case of dissolution profile used directly as input. Green solid line represents the simulated mean PK profile, while the gray solid lines refer to the 5th and 95th simulated percentiles. Colored dots represent the measured observed concentrations at each time point. The insert graph represents a magnification of the main graph for the first 8 h, highlighting absorption.

**Figure 2:**
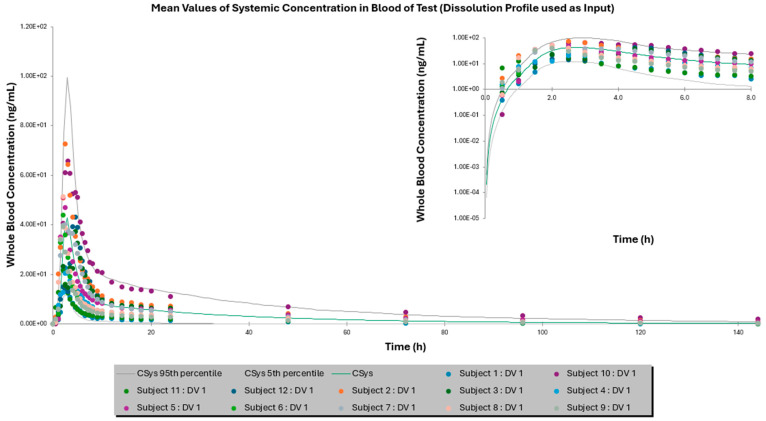
Mean whole-blood in vivo concentration versus time graph of the test formulation after the administration of a single dose for 144 h in the case of dissolution profile used directly as input. Green solid line represents the simulated mean PK profile, while the gray solid lines refer to the 5th and 95th simulated percentiles. Colored dots represent the measured observed concentrations at each time point. The insert graph represents a magnification of the main graph for the first 8 h, highlighting absorption.

Furthermore, Figure 1 and Figure 2 depict the mean whole-blood concentration–time profiles for Advagraf^®^ and the test formulation, respectively, following a single-dose administration over a 144 h period, employing the dissolution profile as a direct input. Corresponding profiles for simulations utilizing the DLM as the input are presented in the Supplementary Materials (Figures S3 and S4). Additionally, dissolution profiles for Advagraf^®^, obtained using different dissolution media in the SIVA toolkit to estimate the relevant DLM scalars, are provided in Figure S1. Respective profiles for the test formulation are also provided in the Supplementary Materials in Figure S2.

Consequently, the two distinct approaches—one employing the dissolution profile as the input and the other utilizing an immediate-release formulation with varying DLM scalars across different gastrointestinal compartments—can be regarded as comparable in terms of in vitro–in vivo extrapolation. Although the analysis was limited to two formulations, namely Advagraf^®^ and the test formulation, the results are promising and highlight the relevance of a biorelevant USP IV dissolution profile for healthy individuals. A notable advantage of the mechanistic approach, which incorporates the immediate-release formulation with varying DLM scalars, is its broader applicability to different populations. For example, physiological changes such as altered mean gastric residence times observed in transplant patients could be accounted for by this methodology, whereas such variability might not be captured when using the dissolution profile directly as the input within the PBPK model. However, implementing this mechanistic approach requires tailored in vitro USP IV dissolution experiments that align with the specific characteristics of each formulation. In certain cases, as exemplified by the test formulation, multiple dissolution experiments may be necessary to comprehensively capture the formulation’s dissolution behavior.

### 3.3. IVIVE to Explore Potential Test Product Candidates for Bioequivalence

**Figure 3:**
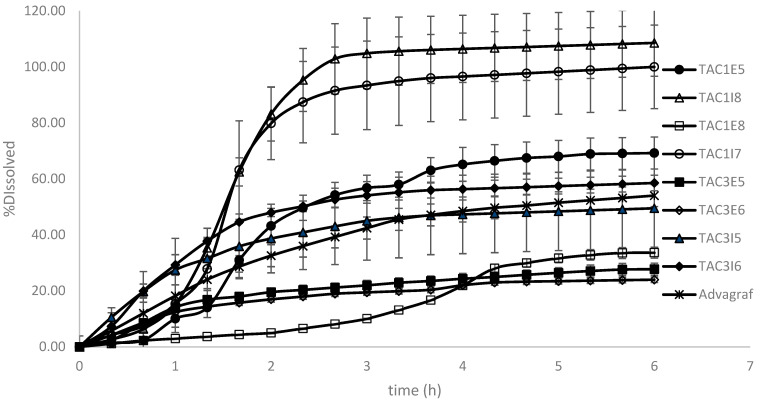
Dissolution profiles for different test formulations and the reference product, Advagraf^®^.

**Figure 4:**
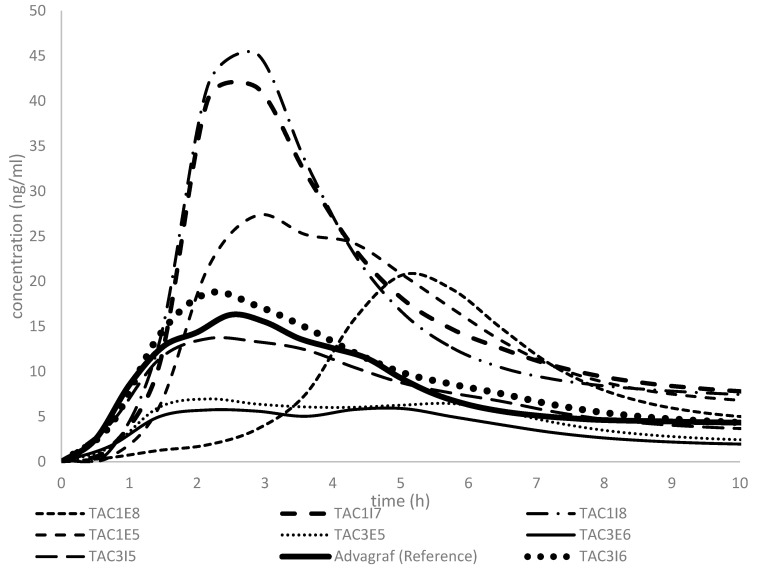
Mean prediction profiles of tacrolimus blood concentrations after the administration of different test formulations and the reference product, Advagraf^®^.

Physiologically based pharmacokinetic (PBPK) modeling has been extensively employed to predict in vivo behavior based on dissolution data. In this study, once the model demonstrated acceptable performance, it was validated and subsequently used to evaluate additional test product formulation candidates for generic development. As previously discussed, seven additional tacrolimus amorphous solid dispersion formulations were prepared and evaluated using the biopredictive dissolution method established earlier. The dissolution profiles for these formulations, presented in Figure 3, were input into the validated PBPK model to predict their respective in vivo concentration–time profiles (Figure 4).

Based on the in vitro behavior of the products, formulations containing Eudragit-S100 tend to exhibit sigmoidal release profiles due to the pH-responsive nature of the polymer, which dissolves at pH levels above 7 [21]. In contrast, formulations containing EC do not exhibit pH dependence, as EC retards release by hindering water penetration [22]. Interestingly, formulations with Eudragit-S100 demonstrate extended dissolution capacity compared to formulations with EC, which exhibit incomplete dissolution. This difference can be attributed to the increased miscibility of tacrolimus with Eudragit-S100 [9], enabling stabilization of the amorphous API and thus preventing recrystallization. Additionally, the mode of surfactant incorporation emerges as a critical factor influencing dissolution behavior. Formulations where the surfactant was incorporated intra-granularly showed more pronounced dissolution. This observation aligns with reports that highlight a stronger surfactant effect when it is intra-granularly incorporated in ASDs [23]. It is generally believed that the intra-granular incorporation of surfactants promotes complexation between the API, polymer, and surfactant, which serves as a mechanism to inhibit crystallization. Furthermore, this mode of surfactant incorporation is thought to retain the surfactant in the vicinity of the dissolving dosage form, thereby facilitating the further solubilization of the API [24]. Finally, visual comparisons indicate that the dissolution behavior of formulations TAC3I5 and TAC3I6 more closely resembles that of the reference product, Advagraf^®^.

These observations are further supported by the PBPK-predicted in vivo concentration–time profiles illustrated in Figure 4. The solid black line represents the reference formulation’s blood concentration–time profile. Notably, formulations containing Eudragit-S100 are projected to exhibit enhanced bioavailability compared to those containing EC. Furthermore, formulations TAC3I5 and TAC3I6 are predicted to closely replicate the shape and bioavailability extent of the reference product curve. This combination of properties makes them promising candidates for generic development. These findings highlight the utility of PBPK modeling in providing valuable insights during the early stages of formulation development. This approach can guide preliminary decisions regarding formulation composition and manufacturing processes, ultimately streamlining the development of generic formulations.

## 4. Conclusions

In this study, PBPK modeling was employed to validate the biorelevance of an in-house dissolution method developed for a controlled-release tacrolimus formulation. A previously published PBPK model was successfully implemented, validated using in-house in vivo data, and subsequently applied to predict in vivo profiles for several tacrolimus formulations under screening. Additionally, the USP IV dissolution method, previously developed, was further assessed as biopredictive, demonstrating its ability to detect formulation differences that translated to in vivo performance. These findings reinforce the critical role PBPK modeling plays in the early stages of generic drug development, particularly in designing biopredictive dissolution methods.

Moreover, formulations with varying composition and manufacturing characteristics were evaluated, allowing key parameters with potential impact on in vivo performance to emerge. Such qualitative, quantitative, and manufacturing insights can significantly streamline the generic development process.

Furthermore, a PBPK model initially developed for an immediate-release (IR) tacrolimus formulation was utilized to establish an IVIVE for a controlled-release product, confirming the validity and transferability of PBPK modeling across diverse applications and conditions. Two different approaches for incorporating dissolution/absorption data were also explored, one using the dissolution profile directly as the input and the other utilizing an IR formulation with varying DLM scalars across gastrointestinal compartments. Both approaches bolstered confidence in the biopredictive capacity of the USP IV dissolution method.

In summary, this work provides compelling evidence of the pivotal role PBPK modeling can play in the early stages of generic drug development, particularly in the design and evaluation of biopredictive dissolution methods.

## Figures and Tables

**Table 1:** Formulation characteristics.

Formulation Code	Polymer Type	Surfactant Type (%)	Surfactant Incorporation Mode
TAC1E5	Eudragit S100	Solutol (0.5%)	extra-granularly
TAC1E8	Eudragit S100	Solutol (3.5%)	extra-granularly
TAC1I8 (Test) *	Eudragit S100	Solutol (3.5%)	intra-granularly
TAC1I7	Eudragit S100	Solutol (0.5%)	intra-granularly
TAC3E5	Ethylcellulose	Soluplus (0.5%)	extra-granularly
TAC3E6	Ethylcellulose	Soluplus (3.5%)	extra-granularly
TAC3I5	Ethylcellulose	Soluplus (0.5%)	intra-granularly
TAC3I6	Ethylcellulose	Soluplus (3.5%)	intra-granularly

* TAC1I8 is denoted as the test formulation.

**Table 2:** Gradient of media introduction in the dissolution cell, along with the flow rates used.

Simulated GI Region	Dissolution Medium	Start Time	Duration	Flow Rate (mL/min)
Stomach	FaSSGF	0	30	8
Proximal gut	FaSSIF-V2	30	40	4
Midgut	FaSSIF-V2 midgut	70	80	4
Distal Ileum	SIF Ileum-V2	150	60	4
Ascending Colon	FaSSCoF	210	150	4

**Table 3:** Mean AUC, Cmax, and Tmax values for observed and simulated PK profiles of both Advagraf^®^ and test formulations, along with the respective fold errors.

	Advagraf^®^			Test Formulation		
	Observed	Simulated with Dissolution Profile	Simulated with DLM	Observed	Simulated with Dissolution Profile	Simulated with DLM *
Mean AUC_0–144 h_ ± SD (ng × h/mL)	355.87 ± 160.22	245.80 ± 177.43	378.12 ± 298.15	462.97 ± 267.64	429.15 ± 380.33	457.86 ± 404.33
Fold Error	-	1.54	1.06	-	1.08	1.01
Mean Cmax ± SD (ng/mL)	16.46 ± 7.18	17.19 ± 11.74	13.10 ± 8.27	43.23 ± 15.54	42.77 ± 28.60	37.03 ± 23.746
Fold Error	-	1.04	1.26	-	1.01	1.17
Mean Tmax ± SD (h)	2.92 ± 1.38	3.05 ± 0.83	3.77 ± 0.9	2.66 ± 0.96	2.83 ± 0.18	2.93 ± 0.51
Fold Error	-	1.04	1.29	-	1.06	1.10

* Unlike other cases, a sensitivity analysis was performed to capture the in vivo PK profile. The parameters that were finally used within SimCyp were DLM scalar = 0.05 in Ileum compartments (I to IV) and DLM scalar = 0.5 in Colon.

**Table 4:** Sensitivity analysis of DLM scalars in Ileum compartments and Colon. In each case, either Ileum DLM scalar, same in all Ileum compartments I to IV, or Colon DLM scalar was kept constant, and mean AUC, Cmax, and Tmax values were recorded.

	Test Formulation with Constant DLM Scalar in Colon, Equal to 0.5			Test Formulation with Constant DLM Scalar in Ileum Compartments (I to IV), Equal to 0.05		
	Ileum DLM Scalar = 0.005	Ileum DLM Scalar = 0.05	Ileum DLM Scalar = 0.5	Colon DLM Scalar = 0.05	Colon DLM Scalar = 0.5	Colon DLM Scalar = 5.0
Mean AUC_0–144 h_ (ng × h/mL)	561.26	457.86	373.52	437.36	457.86	461.22
Mean Cmax (ng/mL)	37.23	37.03	39.53	30.39	37.03	38.61
Mean Tmax (h)	3.81	2.93	2.18	2.82	2.93	2.87

## Data Availability

Datasets are available from the corresponding author upon request.

## Supplementary Materials

The following supporting information can be downloaded at: https://www.mdpi.com/article/10.3390/pharmaceutics17020227/s1, Table S1: SimCYP PBPK and SIVA input parameters from previously published PBPK model [25,26,27,28,29]; Figure S1: % Dissolved over time for Advagraf formulation; Figure S2: % Dissolved over time for Test formulation; Figure S3: Mean whole blood in vivo concentration versus time graph of Advagraf®; Figure S4: Mean whole blood in vivo concentration versus time graph of Test formulation.
