# Supplementary material for: Integrating In Vitro Dissolution and Physiologically Based Pharmacokinetic Modeling for Generic Drug Development: Evaluation of Amorphous Solid Dispersion Formulations for Tacrolimus
Source: Pharmaceutics. 2025 Feb 10;17(2):227. doi: 10.3390/pharmaceutics17020227 (PMC11858865; doi:10.3390/pharmaceutics17020227)
Supplement: Supplementary file 1 [file pharmaceutics-17-00227-s001.zip › pharmaceutics-3463004-supplementary.pdf]

pharmaceutics

Integrating In Vitro Dissolution and Physiologically Based Pharmacokinetic Modeling for Generic Drug Development: Evaluation of Amorphous Solid Dispersion Formulations for Tacrolimus

Evangelos Karakitsios [1,†], Maria-Faidra-Galini Angelerou [2,†], Iasonas Kapralos [1], Georgia Tsakiridou [2], Lida Kalantzi [2] and Aristides Dokoumetzidis [1,*]

[1] Department of Pharmacy, National and Kapodistrian University of Athens, 15771 Athens, Greece; evanskarak@pharm.uoa.gr (E.K.); ikapralo@pharm.uoa.gr (I.K.)
[2] Pharmathen SA, 15125 Athens, Greece; maggelerou@pharmathen.com (M.-F.-G.A.); gtsakiridou@pharmathen.com (G.T.); lkalantzi@pharmathen.com (L.K.)
* Correspondence: adokoum@pharm.uoa.gr
† These authors contributed equally to this work.

Supplementary Information

Table S1: SimCYP PBPK and SIVA input parameters from previously published PBPK model.[15]

**Population**	**Adult Model**	**References**
**Population**	Healthy Volunteers	
**Drug parameters**		
Molecular weight (g/mol)	804.02	[12]
Log Pow	3.26	[12]
Compound type	Neutral	[12]
**Blood binding**		
Concentration-dependent B/P profile		
Bmax (E:P)	80	[25]
KD (μM)	0.004726	[25]
Fraction unbound in plasma (%)	1.2	[26]
**Absorption**		
ADAM model		
Peff (10^{-4} cm/s)	4.77	[27]
DLM model intrinsic solubility (mg/mL)	0.06257	Aqueous buffer solubility for tacrolimus formulation [15]
Logarithm of bile micelle/buffer partitioning coefficient (log Kbmw)	4.55	Fitted using SIVA based on solubility data in acetate buffer, 1/2 FaSSIF, FaSSIF, and FeSSIF [15]
Monodispersed particle size distribution radius (μm)	10	Default SimCyp value
**Distribution**		
Distribution model	Minimal PBPK	[14]

kin (1/h)	0.68	Fitted based on IV data [28]
kout (1/h)	0.12	Fitted based on IV data [28]
Vss (L/kg)	5.5	Fitted based on IV data [28]
kp scalar	10	Fitted based on IV data [28]
Predicted Vss (L/kg)	9.68	Predicted in SimCyp
Prediction method	Method 2	
**Elimination**		
Pathway 1		
CYP3A4		
Vmax (pmol/min/pmol CYP)	8	[29]
Km (μM)	0.21	[29]
CYP3A5		
Vmax (pmol/min/pmol CYP)	17	[29]
Km (μM)	0.21	[29]
Pathway 2		
CYP3A4		
Vmax (pmol/min/pmol CYP)	0.6	[29]
Km (μM)	0.29	[29]
CYP3A5		
Vmax (pmol/min/pmol CYP)	1.4	[29]
Km (μM)	0.35	[29]

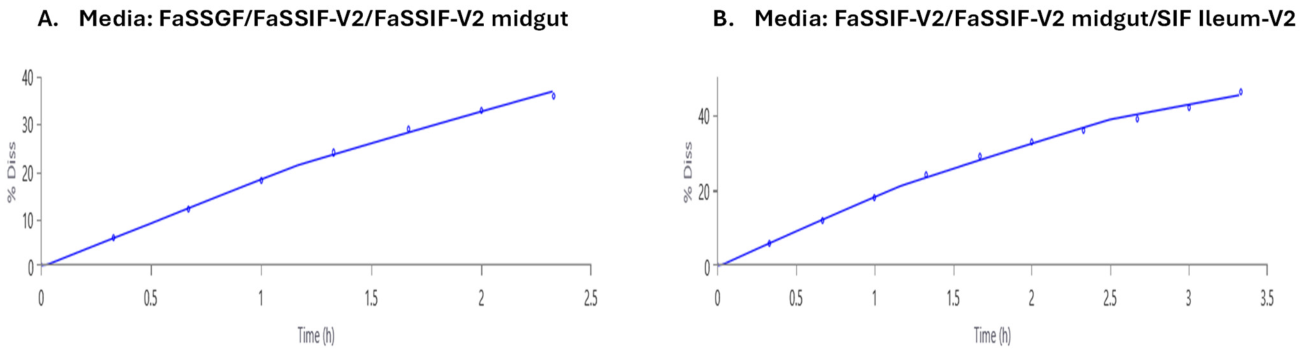

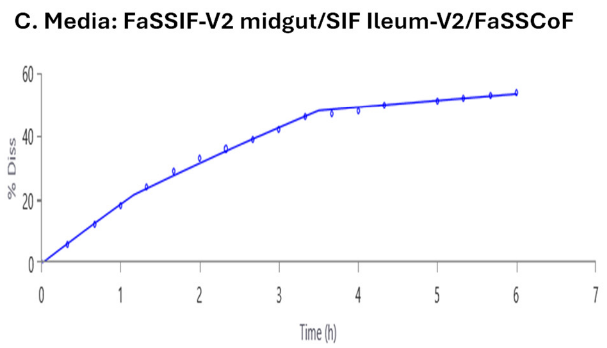

D. Estimated DLM Scalar for each Medium

Medium	DLM Scalar	Relative Standard Error (%)
FaSSGF	0.0345	7.25
FaSSIF-V2	0.0210	7.66
FaSSIF-V2 midgut	0.0279	5.37
SIF Ileum-V2	0.0174	14.7
FaSSCoF	0.00505	14.3

Figure S1: % Dissolved over time for Advagraf formulation utilizing different dissolution media in SIVA toolkit each time to estimate the relevant DLM scalars. A: FaSSGF, FaSSIF-V2 and FaSSIF-V2 midgut were used as dissolution media with the respective experimental in vitro data up to 2.33 hours; B: FaSSIF-V2, FaSSIF-V2 midgut and SIF Ileum-V2 were used as dissolution media with the respective experimental in vitro data up to 3.33 hours; C: FaSSIF-V2 midgut, SIF Ileum-V2 and FaSSCoF were

used as dissolution media with the respective experimental in vitro data up to 6.00 hours; D: Estimated DLM Scalar for each dissolution medium, along with the respective relative standard error.

A. Media: FaSSGF/FaSSIF-V2/FaSSIF-V2 midgut

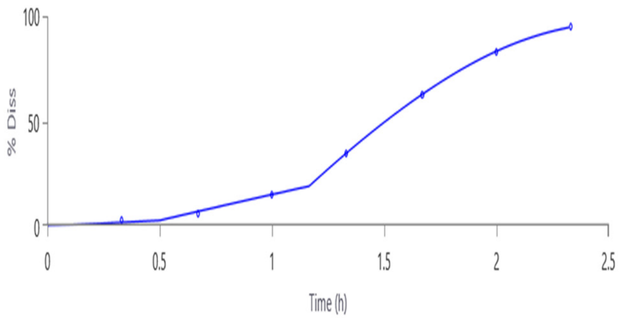

B. Estimated DLM Scalar for each Medium

Medium	DLM Scalar	Relative Standard Error (%)
FaSSGF	0.0102	28.4
FaSSIF-V2	0.0273	7.55
FaSSIF-V2 midgut	0.114	1.73

Figure S2: % Dissolved over time for Test formulation utilizing different dissolution media in SIVA toolkit to estimate the relevant DLM scalars. A: FaSSGF, FaSSIF-V2 and FaSSIF-V2 midgut were used as dissolution media with the respective experimental in vitro data up to 2.33 hours; B: Estimated DLM Scalar for each dissolution medium, along with the respective relative standard error.

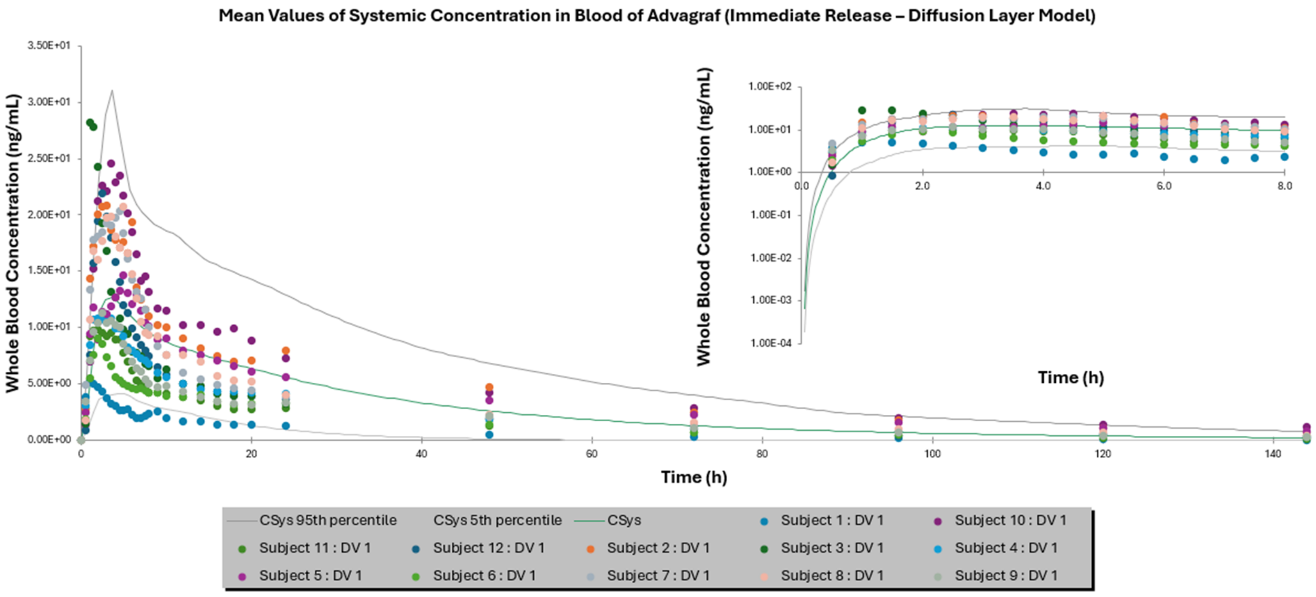

Figure S3: Mean whole blood in vivo concentration versus time graph of Advagraf® after the administration of a single dose for 144 hours, in the case of utilizing immediate release profile and DLM scalars. Green solid line represents the simulated mean PK profile, while the grey solid lines refer to the 5th and 95th simulated percentiles. Colored dots represent the measured observed concentrations at each time point. The insert graph represents a magnification of the main graph for the first 8 hours, highlighting absorption.

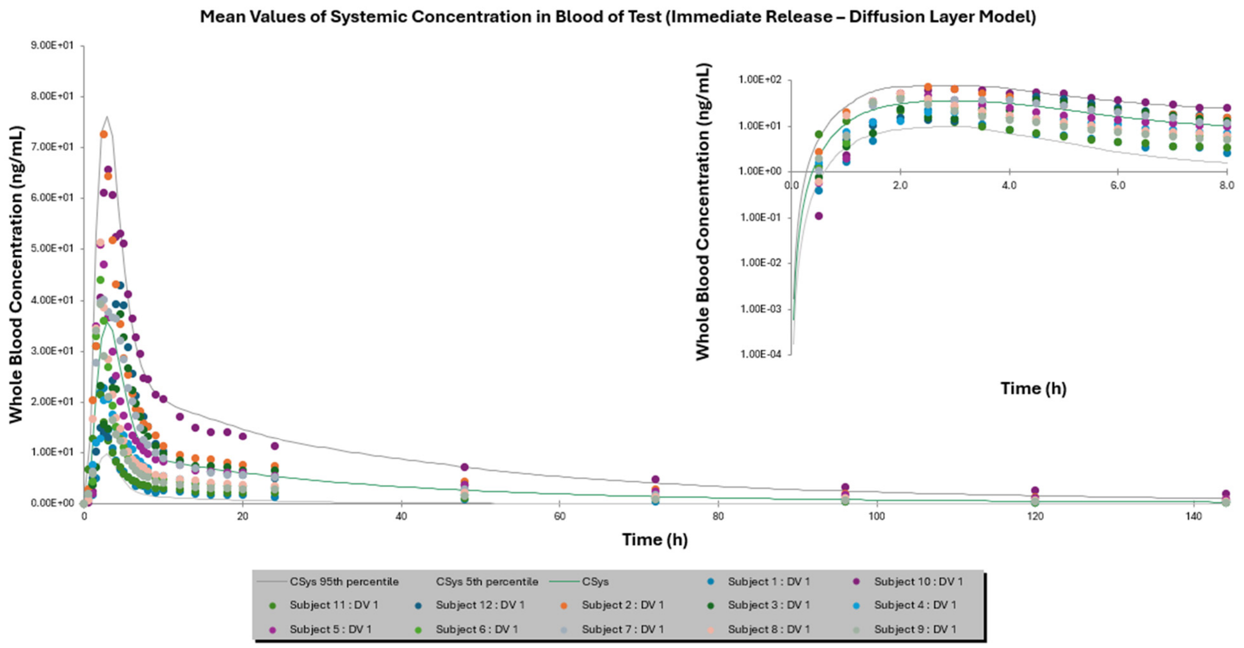

Figure S4: Mean whole blood in vivo concentration versus time graph of Test formulation after the administration of a single dose for 144 hours, in the case of utilizing immediate release profile and DLM scalars. Green solid line represents the simulated mean PK profile, while the grey solid lines refer to the 5th and 95th simulated percentiles. Colored dots represent the measured observed concentrations at each time point. The insert graph represents a magnification of the main graph for the first 8 hours, highlighting absorption. It is noted that DLM scalars that were used, through Sensitivity Analysis, within SimCyp Simulator were DLM scalar = 0.05 in Ileum compartments (I to IV) and DLM scalar = 0.5 in Colon.
